# Applying novel methods in conventional activated sludge plants to treat low-strength wastewater

**DOI:** 10.1007/s10661-022-09968-9

**Published:** 2022-03-31

**Authors:** E. F. Latif

**Affiliations:** Deptartment of Civil Engineering, Faculty of Engineering, Al-Azhar University, Cairo, Egypt

**Keywords:** Conventional activated sludge, Low-strength wastewater, GPS-X simulator, Calibrated model, Filling media

## Abstract

Conventional activated sludge system is confidently widely used for biological treatment plants of municipal wastewater but suffering from operation problems that affect their efficiencies and effluent qualities, especially when treating low-strength wastewater with increasing incoming flow. The objective of this study is to evaluate and compare the novel methods used in upgrading conventional activated sludge treatment systems receiving low-strength wastewater to generate good effluent quality. GPS-X Simulator V 8.0 was used for model calibration and plant performance prediction. The calibrated GPS-X model proved that eliminating primary settling from the treatment process does not affect BOD_5_ and COD removal, while TSS removal is decreased, and NH_4_-N removal is increased. Increasing the return activated sludge flow from 50 to 150% of influent flow does not affect conventional activated sludge process, while the change of waste activated flow had a vital effect on process performance. The presence of an anoxic zone in conventional activated sludge processes treating low-strength wastewater has no significant impact on plant performance. Also, the model outputs proved that adding filling media to the aeration tank was able to handle an increase of influent flow and a stable performance of BOD_5_, and NH_4_-N removal was observed.

## Introduction

Increasing water supply services in developing countries doesn’t meet development in sanitation services, and most of this water directly discharges to water bodies with insufficient or without treatment (Ren et al., 2010). Egypt is suffering from a lack of sanitation services, and most of the generated sewage is directed to surface water bodies (Nasr et al., 2011; States & utvecklingssamarbete, 2013). Egypt issued law 48/1982 and its amendments to regulate the disposal of treated wastewater in the different water bodies (Abdel-Dayem, 2011). Egypt has large centralized wastewater treatment plants (WWTPs) in greater Cairo, ranging from 330,000 m^3^/day in Zenien to 2.5 million m^3^/day in Al-Gabal Al-Asfar, and most of these plants are conventional activated sludge systems (CAS) (Darling & Drake, 1985). The WWTPs are suffering from operation and design problems that affect efficiencies and effluent qualities and do not comply with Egyptian regulations (Wang et al., 2012). All of these WWTPs transfer treated water to surface used water bodies and cause environmental and human health concerns due to the usage of these water (Focazio et al., 2008; Pham & Utsumi, 2018).

The wastewater was characterized as low, medium, and high strength according to the level of contaminations, expressed as chemical oxygen demand, biological oxygen demand, and suspended solids (Metcalf & Eddy, 2014). The municipal wastewater in all of these WWTPs is characterized as medium to low-strength wastewater.

Biological treatment systems have been used for wastewater treatment; conventional activated sludge system confidently is reliably widely used for the biological treatment of municipal wastewater (Hreiz et al., 2015). Sludge bulking was a problem found when treating low-strength wastewater by conventional activated sludge, the low substrate cause sludge flocculent, and predominate filamentous bacteria to cause the sludge bulking phenomenon (Jenkins et al., 2003; Martins et al., 2004; Wei et al., 2015). The major problem in an activated sludge system treating low organic matter concentration is low biomass concentration and poor biomass floc formation in aeration tank, due to insufficient carbonaceous matter for bacterial growth, which leads to a decrease in the removal organic efficiency (Thirumurthi & Orlando, 1976). Also, these WWTPs continuously receive a gradual annual increased incoming flow and contribute to the deterioration of WWTP treated effluents qualities.

The new aerobic granular sludge technology has been used to treat low-strength wastewater (Pronk et al., 2017). Granular aerobic sludge technology, compared with conventional activated sludge, has a compact microbial spherical shape structure, high stability, less sludge production, and good settling ability (Wei et al., 2013). In spite of the advantages of aerobic granular technology, shortage and complexity of substrate associated with low wastewater cause undesirable granular biomass growth. Also, limited research was applied to treat low wastewater, especially in a full-scale plant using aerobic granular technology yet (We et al., 2020). Using granular aerobic sludge technology in existing conventional activated sludge plants is facing the squandering of putting aside both primary and final settling tanks, in addition to the small size of the aeration basin, which isn’t valid to longer operation in sequencing batch reactor mode (Nancharaiah & Reddy, 2018).

Anaerobic wastewater treatment process has been used in treatment of low-strength wastewater (Angenent et al., 2001). The anaerobic treatment systems don’t achieve the desired quality of treatment alone, especially when used for low-strength wastewater treatment (Rodrigues et al., 2001). The problem of odor and clogging associated with anaerobic systems is reducing the tendency to use these technologies especially in inhabited regions and large-scale plants (Manariotis & Grigoropoulos, 2002).

According to the issues stated before, it is needed to develop a novel treatment method applicable in conventional activated sludge system. Mathematical modeling becomes popular in design and simulating the wastewater treatment plant process (Copp et al., 2009). The modeling can easily predict system treatment performance (Ai et al., 2012; Pereira, 2014). So, the objective of this study is to evaluate and compare the novel methods that are used in upgrading conventional activated sludge treatment systems receiving low-strength wastewater to generate good effluent quality comply with environmental regulation and reduce pollution load in the receiving water bodies.

## Materials and methods

### Zenien conventional activated sludge wastewater treatment plant description

**Fig. 1:**
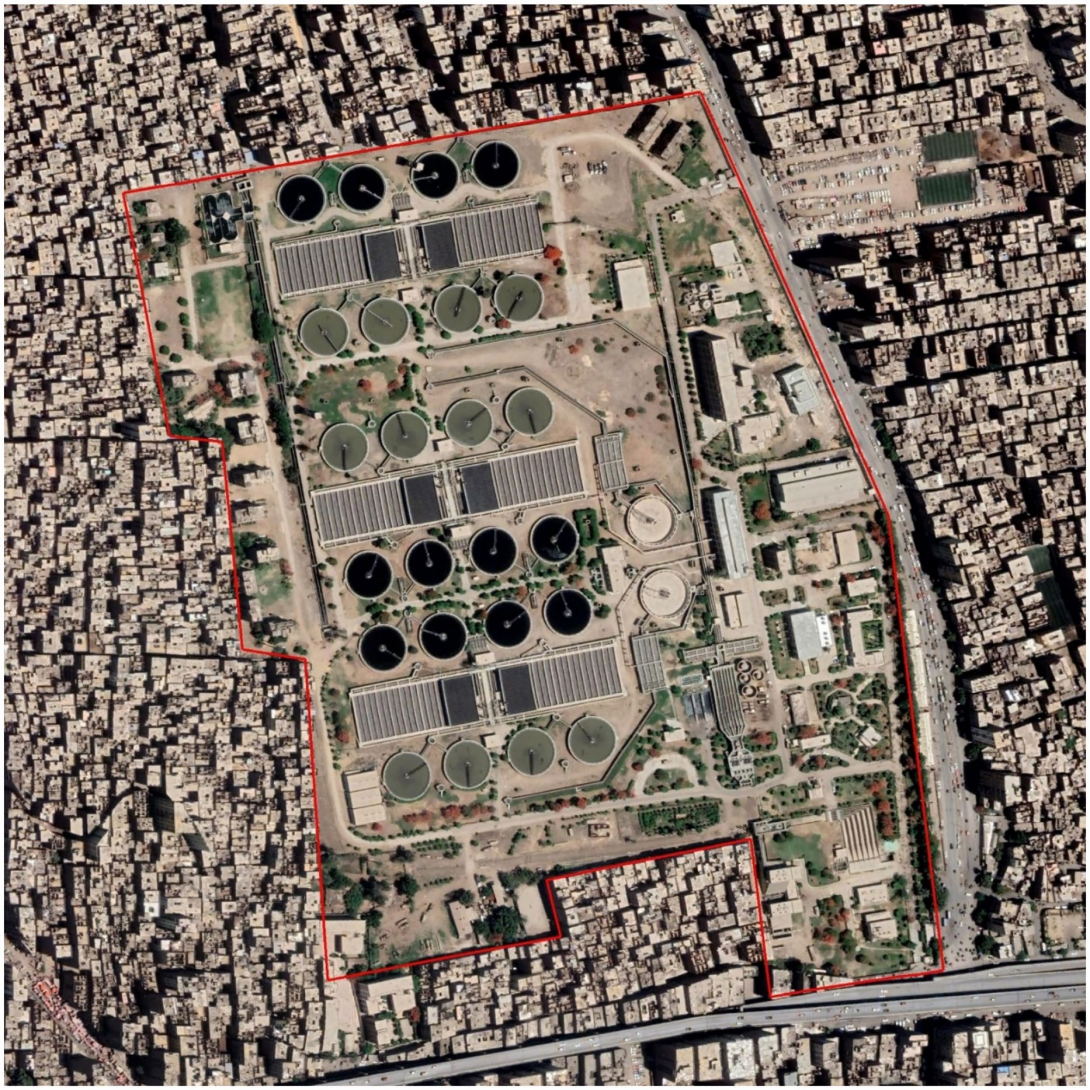
Google Earth photo for Zenien WWTP

The existing Zenien WWTP is a conventional activated sludge system. As shown in Fig. 1 and abridged in Table 1, Zenien WWTP consists of preliminary headworks and three modules for primary and secondary biological treatment; each module consists of four circle primary settling tanks followed by thirty rectangular aeration tanks and then four final settling tanks and contains a return activated pump station. The treated wastewater from all final sedimentation tanks goes towards one chlorine tank and then disposed to agricultural drain, while primary sludge and excess sludge from each module are pumped to one pump station in the plant, which transfers the sludge to handle and dispose it outside, at desert region.

**Table 1:** Abridged data of Zenien WWTP processes units

Unit process	Number	Dimension (m)	Depth (m)
Inlet pump station and inlet chamber	1		
Manual screen	5	L:2	2
Mechanical screen	5	L:2	2
Conventional grit removal chambers	7	L:40 × W:1	3
Preliminary aeration tanks	3	L:40 × W:20	3
Primary settling tanks	12	Ø:36	3
Aeration tanks	90	L:40 × W:5	5
Final settling tanks	12	Ø:36	3
Chlorine contact tank	1	L:30 × W:20	4
Return activated sludge pump station	3		
Excess sludge pump station	3		
Primary sludge pump station	3		
Final sludge pump station	1		
Accessory (chlorination, air blower, transformer, generator, admin, lab, workshop, etc.)	1		

Zenien WWTP is located in Giza governorate, Egypt. Zenien WWTP is constructed in the year 1965 on 96 acres, The design capacity of Zenien WWTP was 330,000 m^3^/day. Now Zenien WWTP receives a flow of 275,000 to 550,000 m^3^/day with an average flow (Q) of 385,000 m^3^/day.

### Wastewater characteristics

The influent and effluent wastewater samples of Zenien municipal WWTP were collected and characterized daily using plant lab staff. The samples were taken from the inlet chamber, final effluent channel, primary settling tank effluent, and final settling tank effluent. The influent and effluent samples were analyzed for total suspended solid (TSS), total chemical oxygen demand (COD), total biochemical oxygen demand (BOD_5_), and ammonia (NH_4_-N). Sludge samples were collected from aeration tanks of three modules for mixed liquor suspended solid (MLSS), mixed liquor volatile suspended solid (MLVSS), and sludge volume index (SVI) measurements. All parameters were measured according to Standard Methods for the Examination of Water and Wastewater (APHA, 2005). Tables 2 to 4 summarize the wastewater average concentration parameters of inlet and outlet processes units in Zenien municipal WWTP. Zenien plant influent wastewater is considered as a low-strength wastewater, where COD, BOD_5_, and TSS concentrations conform to Metcalf and Eddy, (2014) as low-strength wastewater.

This research used the historical wastewater analysis data of Zenien WWTP full-scale conventional activated sludge system, Giza, Egypt, from January to November 2019.

### Modeling Zenien WWTP using GPS-X simulator

Mathematical models were developed to simulate treatment processes in WWTPs and to predict the performance of these plants under different operating conditions (Gernaey et al., 2004; Ji et al., 2019). Various authors use commercial modeling software to study activated sludge wastewater treatment plants, for instance (Abbasi et al., 2021; Latif et al., 2020; Pereira, 2014; Rivas et al., 2008). GPS-X simulator V 8.0 (Hydromantis Environmental Software Solutions, Inc., Canada) is one of the popular modeling software that contains IWA’s activated sludge models and Hydromantis’s models (Hydromantis, 2019). The simulation of Zenien conventional activated sludge WWTP was conducted using GPS-X Simulator V 8.0 with the help of GPS-X manuals.

**Fig. 2:**
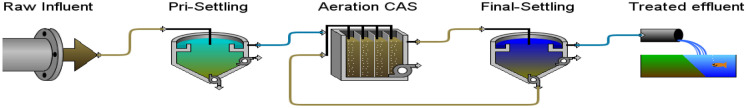
Zenien CAS WWTP layout in GPS-X simulator

Zenien WWTP model construction in GPS-X was based on the plant’s real municipal wastewater characteristics and actual operation data presented previously for 11 months. The model as shown in Fig. 2 consists of raw influent wastewater, primary settling tank, aeration tank, final settling tank, and treated wastewater effluent blocks. The plant flow was divided by twelve to simulate one primary and final sedimentation tank, while this ratio was converted to be used in one aeration tank dimensions. The influent block was used for raw wastewater characteristics; the physical dimensions and operation condition of the plant were assigned as actual plant data in primary, final sedimentation tanks, and aeration tanks, while the effluent block represented treated wastewater outfall. The biological model used to simulate the Zenien WWTP was Hydromantis’s Mantis2, which was used for the organic and nutrient simulation process (Hydromantis, 2019), and the simulation results were obtained using a default kinetics parameter of the model.

### Calibration and validation of GPS-X model

The simulation results of Zenien WWTP model in GPS-X were compared with the actual plant results in Tables 2, 3, and 4. The GPS-X Zenien model was calibrated and validated to allow the simulated results to fit the actual plant results (Abou-Elela et al., 2016), in which the reliance was 95%, using one-way ANOVA statistical between real and simulated results (Mu’azu et al., 2020).

**Table 2:** Characteristic of influent and effluent wastewater for primary treatment in Zenien WWTP

Month	1		2		3		4		5		6
Parameter	Influent	Effluent	Influent	Effluent	Influent	Effluent	Influent	Effluent	Influent	Effluent	Influent
TSS, mg/L	130 ± 16	80 ± 19	135 ± 24	76 ± 11	133 ± 34	76 ± 13	125 ± 27	74 ± 21	131 ± 38	83 ± 14	132 ± 21
BOD_5_, mg/L	138 ± 26	76 ± 27	137 ± 22	66 ± 14	133 ± 16	68 ± 17	127 ± 26	86 ± 23	136 ± 20	90 ± 28	128 ± 20
COD, mg/L	274 ± 54	164 ± 31	309 ± 45	185 ± 30	302 ± 43	181 ± 20	296 ± 50	200 ± 18	335 ± 70	222 ± 24	293 ± 75

Month	6	7		8		9		10		11	
Parameter	Effluent	Influent	Effluent	Influent	Effluent	Influent	Effluent	Influent	Effluent	Influent	Effluent
TSS, mg/L	75 ± 22	124 ± 44	63 ± 16	118 ± 27	69 ± 14	134 ± 20	68 ± 22	127 ± 34	68 ± 20	147 ± 34	76 ± 27
BOD_5_, mg/L	81 ± 27	104 ± 27	73 ± 20	114 ± 25	74 ± 15	129 ± 18	77 ± 19	132 ± 33	77 ± 19	129 ± 34	76 ± 21
COD, mg/L	185 ± 24	230 ± 48	161 ± 28	256 ± 50	166 ± 34	290 ± 50	173 ± 24	320 ± 58	187 ± 17	293 ± 50	173 ± 32

**Table 3:** Characteristic of influent and effluent wastewater for secondary treatment in Zenien WWTP

Month	1		2		3		4		5		6
Parameter	Influent	Effluent	Influent	Effluent	Influent	Effluent	Influent	Effluent	Influent	Effluent	Influent
TSS, mg/L	80 ± 19	26 ± 7	76 ± 11	22 ± 6	76 ± 13	18 ± 4	74 ± 21	14 ± 5	83 ± 14	15 ± 7	75 ± 22
BOD5, mg/L	76 ± 27	19 ± 7	66 ± 14	16 ± 5	68 ± 17	15 ± 5	86 ± 23	12 ± 5	90 ± 28	13 ± 5	81 ± 27
COD, mg/L	164 ± 31	59 ± 13	185 ± 30	55 ± 4	181 ± 20	53 ± 9	200 ± 18	54 ± 9	222 ± 24	54 ± 17	185 ± 24
NH_4_-N, mg/L	19.5 ± 3	9.9 ± 2	21.5 ± 4	8.8 ± 2	18.7 ± 1	7.3 ± 1	21.3 ± 2	10.5 ± 2	18.4 ± 3	10.9 ± 2	18.9 ± 1

Month	6	7		8		9		10		11	
Parameter	Effluent	Influent	Effluent	Influent	Effluent	Influent	Effluent	Influent	Effluent	Influent	Effluent
TSS, mg/L	18 ± 4	63 ± 16	12 ± 4	69 ± 14	15 ± 4	68 ± 22	15 ± 6	68 ± 20	15 ± 9	76 ± 27	18 ± 8
BOD5, mg/L	16 ± 4	73 ± 20	11 ± 4	74 ± 15	14 ± 3	77 ± 19	15 ± 6	77 ± 19	15 ± 7	76 ± 21	17 ± 8
COD, mg/L	52 ± 12	161 ± 28	43 ± 10	166 ± 34	38 ± 8	173 ± 24	39 ± 6	187 ± 17	32 ± 16	173 ± 32	42 ± 14
NH_4_-N, mg/L	10.2 ± 2	18.1 ± 3	9.1 ± 2	17.5 ± 3	9.4 ± 3	18.6 ± 4	9.5 ± 3	16.6 ± 3	6.6 ± 4	23.5 ± 3	8.8 ± 4

**Table 4:** Parameters of aeration tank in Zenien WWTP

Month	1	2	3	4	5	6	7	8	9	10	11
Food to microorganism ratio (F/M), 1/d	0.3 ± 0.1	0.2 ± 0.1	0.2 ± 0.1	0.4 ± 0.1	0.3 ± 0.1	0.4 ± 0.1	0.4 ± 0.1	0.4 ± 0.1	0.4 ± 0.1	0.4 ± 0.1	0.3 ± 0.1
SVI, ml/g	158 ± 48	155 ± 42	159 ± 51	175 ± 56	176 ± 64	176 ± 58	188 ± 63	198 ± 65	179 ± 60	161 ± 51	141 ± 46
MLSS mg/L	1846 ± 506	1660 ± 397	1519 ± 342	1452 ± 400	1253 ± 333	1424 ± 290	1229 ± 306	1155 ± 260	1246 ± 275	1302 ± 304	1377 ± 400
MLVSS, mg/L	1605 ± 441	1449 ± 345	1327 ± 297	1303 ± 357	1146 ± 303	1255 ± 256	1068 ± 266	1024 ± 230	1069 ± 234	1118 ± 259	1174 ± 341
Solid retention time (SRT), d	4 ± 2	5 ± 2	5 ± 2	5 ± 2	7 ± 2	4 ± 2	5 ± 2	5 ± 2	5 ± 2	5 ± 2	6 ± 2
*Flow, m^3^/d × 1000	381 ± 19.2	370 ± 28.7	372 ± 23.3	382 ± 21	356 ± 31.6	401 ± 73.8	412 ± 26.5	394 ± 23.9	383 ± 33.1	402 ± 31.6	391 ± 50
Return activated sludge (RAS), % of flow	23 ± 2	23 ± 1	23 ± 2	23 ± 2	23 ± 2	25 ± 3	23 ± 1	24 ± 2	23 ± 3	22 ± 2	22 ± 3
Waste activated sludge (WAS), % of flow	0.5 ± 0.2	0.5 ± 0.1	0.4 ± 0.1	0.6 ± 0.2	0.5 ± 0.2	0.5 ± 0.1	0.5 ± 0.2	0.5 ± 0.1	0.5 ± 0.1	0.4 ± 0.1	0.3 ± 0.2
Dissolved Oxygen (DO), mg/L	1.6 ± 0.5	1.3 ± 0.6	1.3 ± 0.5	1.3 ± 0.5	1.2 ± 0.4	1.2 ± 0.4	1.5 ± 0.7	1.6 ± 0.7	1.5 ± 0.8	1.9 ± 0.8	1.7 ± 0.6
*Temperature (T), ^0^C	18 ± 2	19 ± 2	20 ± 2	23 ± 2	26 ± 2	27 ± 3	30 ± 3	30 ± 2	26 ± 2	25 ± 2	23 ± 2
*pH	7.1 ± 0.3	7.2 ± 0.2	7.2 ± 0.3	7.2 ± 0.2	7.2 ± 0.2	7.1 ± 0.3	7.2 ± 0.2	7.2 ± 0.2	7.2 ± 0.3	7.3 ± 0.3	7.3 ± 0.2

*They are measured and recorded for influent flow

### Applying novel treatment methods in Zenien conventional activated sludge plant

Calibrated GPS-X model of Zenien CAS WWTP was used for applying a novel treatment method for studying and enhancement of plant performance. Through the calibrated model, GPS-X was used to create five scenarios: (a) bypassing primary settling from plant process, (b) effect of return activated sludge (Qr) and waste sludge (Qw) flows, (c) effect of anoxic/oxic (A/O) zones, (d) effect of adding media to the aeration tank; and (e) effect of increasing influent flow.

## Results and discussions

### Zenien CAS WWTP model calibration and validation

The simulation results of Zenien WWTP model using GPS-X simulator were obtained by default kinetics parameter of the model; we found that the simulation results are different from the real plant result effluents in terms of COD, BOD_5_, and NH_4_-N; thus, the model calibration was essential.

The most sensitive kinetic parameters of the biological model were investigated by the researchers, Latif et al. (2020) who found a change of aerobic heterotrophic yield (Y_H_) and aerobic heterotrophic decay rate (b_H_) values, which affect the model in terms of COD and BOD_5_ effluent concentrations; change maximum growth rate for ammonia oxidizer (μ_A_), ammonia oxidizer aerobic decay rate (b_A_), and oxygen saturation for ammonia oxidizer (K_O_) values; and affect the model in term NH_4_-N effluent concentration. Moretti et al. (2018) found (μ_A_), (b_A_), and (K_O_) were most sensitive model parameter for ammonia removal. Liwarska-Bizukojc et al. (2011) found (μ_A_), (b_A_), and (K_O_); substrate (NH_4_-N) half-saturation constant (K_NH4_); maximum growth rate of heterotrophic organisms (μ_H_); and substrate (COD) half-saturation constant (K_S_), (b_H_), and (Y_H_) were sensitively model parameters for COD, BOD_5_, and NH_4_-N removals. Hu et al. (2014) found (μ_H_), (Y_H_) (K_S_), (b_H_), (μ_A_), (K_NH4_), and (b_A_) were sensitive parameters related to COD and NH_4_-N effluent concentrations.

The used Hydromantis’s Mantis2 model contains over 50 kinetic and stoichiometric parameters. In order to conduct sensitivity analysis in GPS-X simulator for the determination of sensitive parameters that will be used for model calibration, it will take a long time so, based on the most sensitive parameters that were found in the literature (μ_H_, Y_H_, b_H_, μ_A_, K_O_, b_A_, and K_NH4_) which were considered sensitively acceptable parameters used in model calibration.

**Fig. 3:**
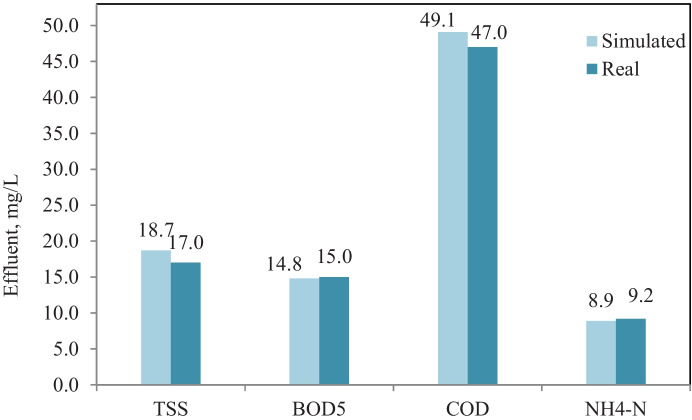
Average effluent concentration of real and calibrated model simulated results

Model calibration and validation in GPS-X simulator were performed by adjusting model kinetic parameters; Table 5 shows kinetic parameters (default, adjusted, and reported in the literature) values. Figure 3 shows an average effluent concentration of real plant results versus calibrated model results, where the model results fit with real plant results in terms of TSS, BOD_5_, COD, and NH_4_-N effluent concentrations. The results demonstrate validation of the model comparing with real plant results through eleven months, which no significant difference between simulated and real results for TSS, BOD_5_, COD, and NH_4_-N effluent concentrations (Petersen et al., 2003).

**Table 5:** Calibrated model kinetic parameters and the reported range in the literature

Stoichiometric/kinetic parameters	Default	Range	Reference	Calibrated values (this study)
*μ*_H_	3.2	0.6–13.2	(Hydromantis, 2019)	2.20
*Y*_H_	0.666	0.38–0.75	(Jeppsson, 1996)	0.45
*b*_H_	0.62	0.05–1.6	(Hydromantis, 2019; Mulas, 2006)	1.30
*μ*_A_	0.90	0.2–1.2	(Afonso & da Conceição Cunha, 2002; Soliman & Eldyasti, 2018)	0.56
*K*_o_	0.25	0.1–1	(Boontian, 2012; Jeppsson, 1996; Soliman & Eldyasti, 2018)	0.75
*b*_A_	0.17	0.05–0.3	(Jeppsson, 1996; Soliman & Eldyasti, 2018; Weijers & Vanrolleghem, 1997)	0.27
*K*_NH4_	0.70	0.14–1.35	(Soliman & Eldyasti, 2018)	1.0

### Applying novel treatment methods in Zenien conventional activated sludge plant

#### Bypassing primary settling from plant process scenario

**Fig. 4:**
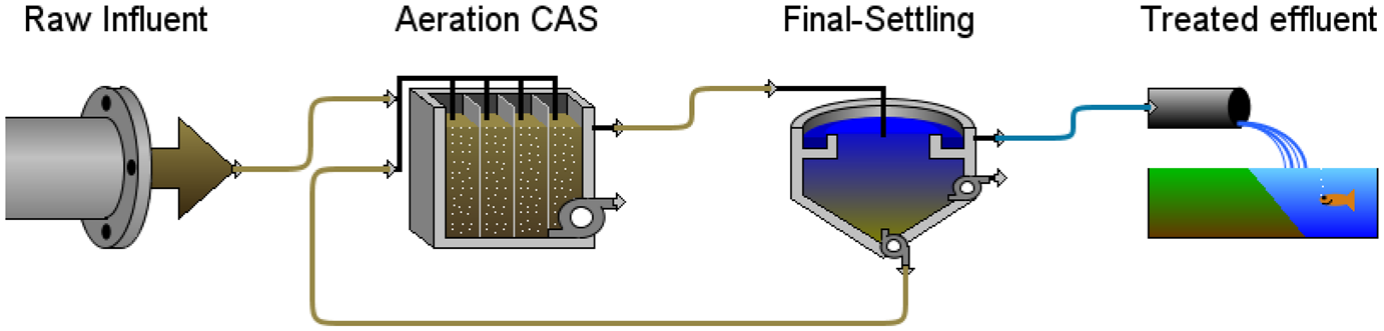
Bypassing primary settling from the plant process scenario layout in GPS-X simulator

**Fig. 5:**
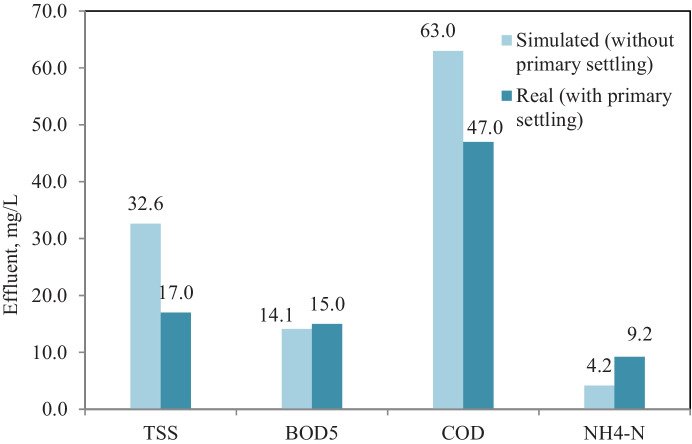
Simulation results of bypassing primary settling from plant process scenario and real plant results with primary settling

Bypassing primary settling from the plant process scenario was simulated using the calibrated model to predict and test the plant performance without primary settling. Figure 4 shows the flow diagram of bypassing primary settling from plant process scenario in GPS-X simulator. Figure 5 shows effluent concentrations of TSS, BOD_5_, COD, and NH_4_-N corresponding to removal efficiency of 74.3, 89, 78.3, and 78.3%, respectively, in case of plant without primary settling and no significant difference between removal efficiency for BOD_5_ and COD with and without primary settling, in which the average removal efficiency of BOD_5_ and COD was 88.4 and 83.7% at real record results of plant with primary settling. TSS removal efficiency decreased from 86.6 to 74.3% and NH_4_-N removal efficiency increased from 52.5 to 78.3%, clearly significant between with and without primary settling scenarios. The decrease of TSS removal may be ascribed to the elimination of primary settling, while the increase of ammonia removal may be ascribed to increase nitrifier bacteria, due to an increase of biomass concentration in aeration tank with the elimination of primary settling (Gerardi, 2011).

#### Effect of return activated sludge and excess sludge flows scenario

**Fig. 6:**
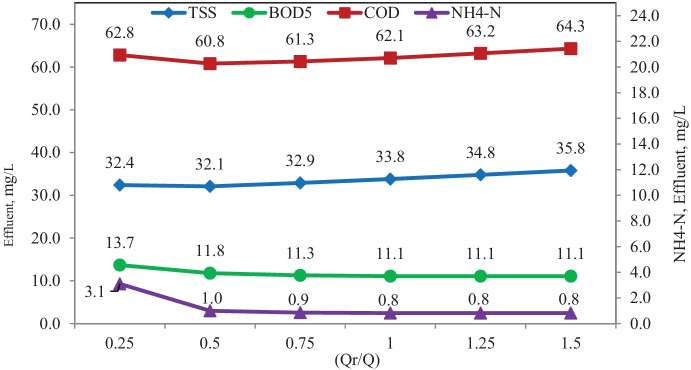
Simulation results of the effect of return activated sludge scenario

A study of the effect of return activated sludge and excess sludge flows scenario was carried out also without primary settling using the same flow diagram shown in Fig. 4. Figure 6 shows the effect of return activated sludge percent in terms of TSS, BOD_5_, COD, and NH_4_-N effluent concentrations. The simulation results of this scenario proved that no significant difference between TSS, BOD_5_, COD, and NH_4_-N effluent concentration with the change of return activated sludge percentage between 0.25 and 1.5 of influent flow, except ammonia at 0.25 value, which has a significant removal decrease than other values, and this could be ascribed to lower nitrifier bacteria concentration at 0.25 value than other values. The Qr/Q of 0.5 is considered optimum from the economic view.

Study of the effect of waste sludge flow scenario was carried out also without primary settling at 50% return activated sludge flow. The waste sludge flows were 50, 100, 150, 200, 250, 300, 350, and 400 m^3^/day corresponding to (Qw/Qr) of 0.31, 0.63, 0.94, 1.25, 1.56, 1.88, 2.19, and 2.5% and SRT of 16, 15, 14, 12, 11, 9, 8, and 7 days, respectively.

**Fig. 7:**
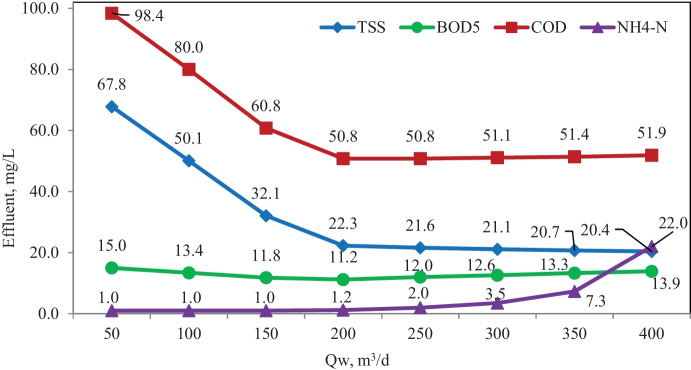
Simulation results of the effect of waste sludge scenario

Figure 7 shows the effect of waste sludge flow in terms of TSS, BOD_5_, COD, and NH_4_-N effluent concentrations. The simulation results of this scenario proved that variation of waste sludge between 50 and 400 m^3^/day doesn’t significantly affect BOD_5_ removal.

Increased waste flow from 50 to 200 m^3^/day significantly improved COD and TSS removal, where no significant effect was observed when waste flow increased up to 400 m^3^/day.

Increased waste flow from 50 to 200 m^3^/day does not significantly affect NH_4_-N removal, while increasing the waste flow to 400 m^3^/day significantly decreases NH_4_-N removal.

The optimum value for excess sludge flow is considered 200 m^3^/day with regard to the economic and removal view.

#### Effect of introducing anoxic zone scenario

**Fig. 8:**
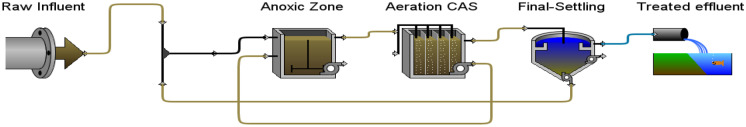
Anoxic/aeration zone scenario layout in GPS-X simulator

The effect of introducing an anoxic zone with internal recycling on conventional activated sludge process was tested using calibrated GPS-X model. Figure 8 shows the flow diagram of anoxic/oxic zones scenario in GPS-X simulator. Primary settling was bypassed, internal recycle was 200% of influent flow, the anoxic zone was 25% of aeration tank volume, waste sludge flow was 200 m^3^/day, and return activated sludge percent was 50% of the influent flow.

**Fig. 9:**
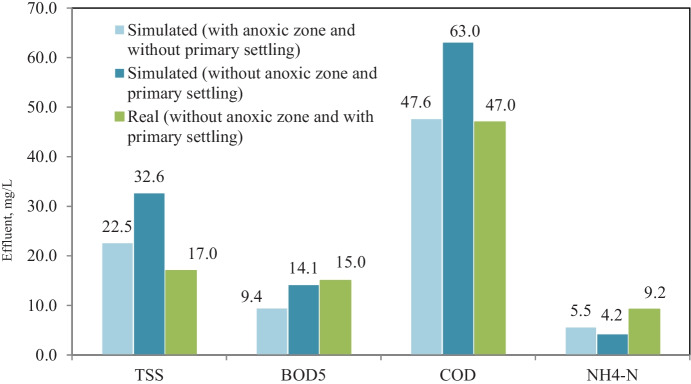
Simulation results of the effect of alternate aeration tank to anoxic/oxic zones scenario

Figure 9 shows effluent concentrations of TSS, BOD_5_, COD, and NH_4_-N; the results prove no significant removal rate between removal efficiency for BOD_5_ and COD with and without the anoxic zone in case of bypassing primary settling, while TSS removal efficiency significantly increased, and NH_4_-N removal efficiency significantly decreased. It is concluded that introducing an anoxic zone to the conventional activated sludge process improves TSS, BOD_5_, and COD removal and slightly decreases the NH_4_-N removal in case of bypassing primary settling and no significant impact TSS, BOD_5_, and COD removal in case of existing the primary settling.

#### Effect of filling media scenario

**Fig. 10:**
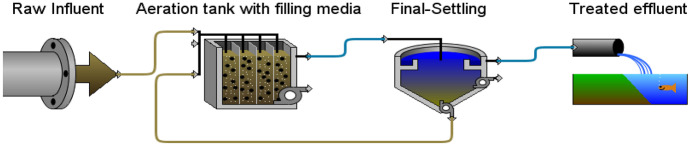
Aeration tank with filling media scenario layout in GPS-X simulator

The effect of filling aeration tank with plastic media was investigated using calibrated GPS-X model. Primary settling was bypassed, waste sludge was 200 m^3^/day, return activated sludge percent was 50% of influent flow, the filling ratio was 50% of aeration tank volume, and media surface area was 500 m^2^/m^3^. Figure 10 shows the flow diagram of aeration tank with filling media scenario in GPS-X simulator.

**Fig. 11:**
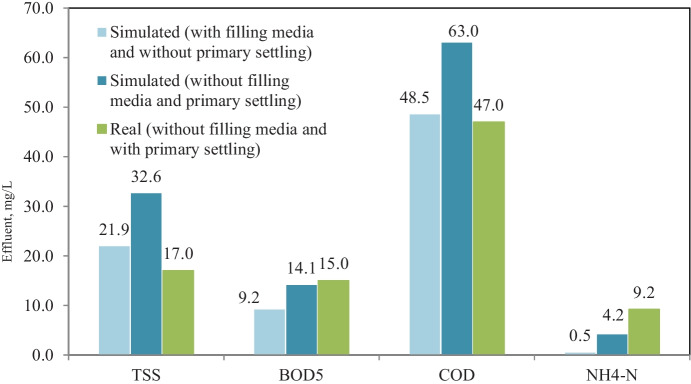
Simulation results of adding media to aeration tank scenario

Figure 11 shows effluent concentrations of TSS, BOD_5_, COD, and NH_4_-N; the results prove no significant difference between removal efficiency for BOD_5_ and COD with and without filling media in case of bypassing primary settling, while TSS and NH_4_-N removal efficiency significantly increased. It is concluded that adding filling media to aeration tank of conventional activated sludge process significantly improves NH_4_-N removal.

#### Effect of increasing influent flow scenario

**Fig. 12:**
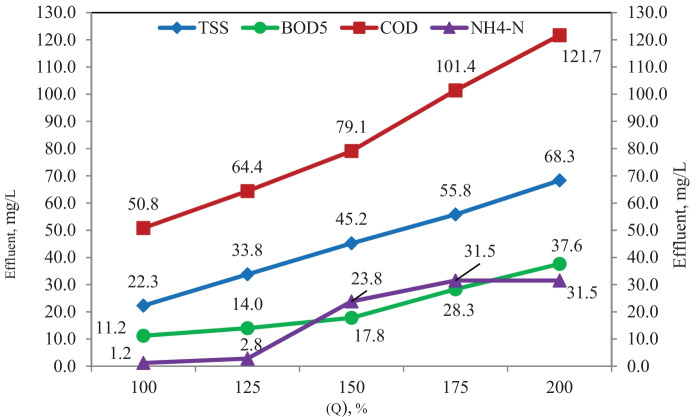
Simulation results of the effect of the increased influent flow without filling media scenario

**Fig. 13:**
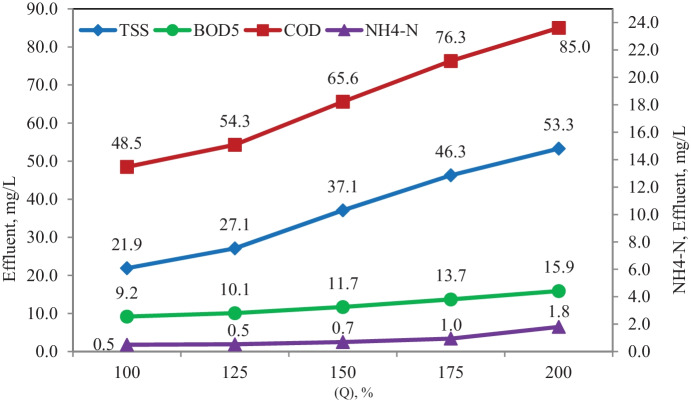
Simulation results of the effect of the increased influent flow with filling media scenario

The effect of increasing influent on aeration tank was investigated as shown in Figs. 12, 13; the influent flow was increased from 100 to 200%, applying to aeration tank in the previous scenarios (with and without adding media) using the same flow diagram shown in Fig. 10. It was proved, as shown in Fig. 12, that increasing influent flow from 125 to 200% significantly decreases removal of TSS, BOD_5_, COD, and NH_4_-N, and this decrease is ascribed to low hydraulic retention time with the increasing influent flow (Aghapour et al., 2013; Sirianuntapiboon et al., 2005). As shown in Fig. 13, it was proved that increasing influent flow had no significant effect in terms of BOD_5_ and NH_4_-N removal; this may be ascribed to the activity of biomass attached to the media in aeration tank that supports the addition of both heterotrophic and nitrifier bacteria (Randall & Sen, 1996; Sirianuntapiboon & Yommee, 2006), but a significant effect in terms of TSS and COD removal was observed, and this could be ascribed to complex degradation of low-strength substrate (Mannina et al., 2017).

## Conclusions

- The calibrated GPS-X model was used to predict the effect of eliminating primary settling from conventional activated sludge process when treating low-strength wastewater. The model outputs proved that eliminating primary settling from the treatment process did not affect BOD_5_ and COD removal, while TSS removal is decreased, and NH_4_-N removal is increased.
- The model outputs proved that increasing the return activated sludge flow from 50 to 150 of influent flow didn’t affect conventional activated sludge process, while change waste activated flow had a vital effect on process performance.
- The model outputs proved that the presence of anoxic phase in conventional activated sludge process treating low-strength wastewater has no significant impact on plant performance
- The model outputs proved that adding filling media to the aeration tank in conventional activated sludge process treating low-strength wastewater will enable to handle increase influent flow with stable performance for BOD_5_ and NH_4_-N removal, as was observed, rather than the system without media.

## Data Availability

All data analyzed during this study are included in this article. The raw data that support the findings of this study are available on request from the author.
