# Correction to: Applying novel methods in conventional activated sludge plants to treat low‑strength wastewater

**DOI:** 10.1007/s10661-024-12828-3

**Published:** 2024-06-24

**Authors:** E. F. Latif

**Affiliations:** Department of Civil Engineering, Faculty of Engineering, Al-Azhar University, Cairo, Egypt

**Correction to: Environ Monit Assess (2022) 194: 323**

10.1007/s10661-022-09968-9

The original version of this article unfortunately contained an error in the affiliation section.

The incorrect/misspelled word “Deptartment” in the affiliation section should be corrected from "Deptartment of Civil Engineering" to “Department of Civil Engineering”.

The affiliation section has been corrected.

